# Addressing inter individual variability in CSF levels of brain derived proteins across neurodegenerative diseases

**DOI:** 10.1038/s41598-024-83281-y

**Published:** 2025-01-03

**Authors:** Sára Mravinacová, Sofia Bergström, Jennie Olofsson, Nerea Gómez de San José, Sarah Anderl-Straub, Janine Diehl-Schmid, Klaus Fassbender, Klaus Fliessbach, Holger Jahn, Johannes Kornhuber, G. Bernhard Landwehrmeyer, Martin Lauer, Johannes Levin, Albert C. Ludolph, Johannes Prudlo, Anja Schneider, Matthias L. Schroeter, Jens Wiltfang, Petra Steinacker, FTLD Consortium, Sarah Anderl-Straub, Janine Diehl-Schmid, Klaus Fassbender, Klaus Fliessbach, Holger Jahn, Johannes Kornhuber, G. Bernhard Landwehrmeyer, Martin Lauer, Johannes Levin, Albert C. Ludolph, Johannes Prudlo, Anja Schneider, Matthias L. Schroeter, Jens Wiltfang, Petra Steinacker, Markus Otto, Markus Otto, Peter Nilsson, Anna Månberg

**Affiliations:** 1 Department of Protein Science, SciLifeLab, KTH Royal Institute of Technology, Stockholm, Sweden; 2 Department of Neurology, University Hospital Ulm (UKU), Ulm, Germany; 3 Department of Psychiatry, Technical University of Munich, Munich, Germany; 4 Kbo-Inn-Salzach-Klinikum Gemeinnützige GmbH, Wasserburg Am Inn, Germany; 5 Department of Neurology, Saarland University, Homburg, Germany; 6 Department of Neurodegenerative Diseases and Geriatric Psychiatry, University of Bonn and DZNE Bonn, Bonn, Germany; 7 Department of Psychiatry, University Hospital, Hamburg, Germany; 8 Department of Psychiatry, Friedrich-Alexander University Erlangen-Nuremberg, Erlangen, Germany; 9 Center for Mental Health, Department of Psychiatry, Psychosomatics and Psychotherapy, University Hospital Würzburg, Würzburg, Germany; 10 Department of Neurology, LMU University Hospital, LMU Munich, Munich, Germany; 11 German Center for Neurodegenerative Diseases, Site Munich, Munich, Germany; 12 Munich Cluster for Systems Neurology (SyNergy), Munich, Germany; 13 German Center for Neurodegenerative Diseases (DZNE E.V.), Ulm, Germany; 14 Rostock University Medical Center and German Center for Neurodegenerative Diseases (DZNE), Rostock, Germany; 15 Clinic for Cognitive Neurology, University Clinic Leipzig, and Max Planck Institute for Human Cognitive and Brain Sciences, Leipzig, Germany; 16 Department of Psychiatry and Psychotherapy, University Medical Center Goettingen, and DZNE, Goettingen, Germany; 17 Neurosciences and Signaling Group, Institute of Biomedicine (iBiMED), Department of Medical Sciences, University of Aveiro, Aveiro, Portugal; 18 Department of Neurology, Martin-Luther-University Halle-Wittenberg, Halle (Saale), Germany

**Keywords:** Inter-individual variability, Affinity proteomics, Biomarker, Cerebrospinal fluid, Multi-disease, Neurodegeneration, Molecular neuroscience, Diagnostic markers, Proteomics

## Abstract

Accurate diagnosis and monitoring of neurodegenerative diseases require reliable biomarkers. Cerebrospinal fluid (CSF) proteins are promising candidates for reflecting brain pathology; however, their diagnostic utility may be compromised by natural variability between individuals, weakening their association with disease. Here, we measured the levels of 69 pre-selected proteins in cerebrospinal fluid using antibody-based suspension bead array technology in a multi-disease cohort of 499 individuals with neurodegenerative disorders including Alzheimer’s disease (AD), behavioral variant frontotemporal dementia, primary progressive aphasias, amyotrophic lateral sclerosis (ALS), corticobasal syndrome, primary supranuclear palsy, along with healthy controls. We identify significant inter-individual variability in overall CSF levels of brain-derived proteins, which could not be attributed to specific disease associations. Using linear modelling, we show that adjusting for median CSF levels of brain-derived proteins increases the diagnostic accuracy of proteins previously identified as altered in CSF in the context of neurodegenerative disorders. We further demonstrate a simplified approach for the adjustment using pairs of correlated proteins with opposite alteration in the diseases. With this approach, the proteins adjust for each other and further increase the biomarker performance through additive effect. When comparing the diseases, two proteins—neurofilament medium and myelin basic protein—showed increased levels in ALS compared to other diseases, and neurogranin showed a specific increase in AD. Several other proteins showed similar trends across the studied diseases, indicating that these proteins likely reflect shared processes related to neurodegeneration. Overall, our findings suggest that accounting for inter-individual variability is crucial in future studies to improve the identification and performance of relevant biomarkers. Importantly, we highlight the need for multi-disease studies to identify disease-specific biomarkers.

## Introduction

Neurodegenerative disorders encompass a variety of diseases characterised by a gradual loss of neurons. The aetiology of such diseases is not yet fully understood. However, pathological mechanisms such as abnormal protein aggregations and neuroinflammation are commonly involved^1,2^. The continuously increasing prevalence, associated burden of deaths, and rising costs underscore the recognition of neurodegenerative disorders as a major public health challenge^3^. Dementias represent a large proportion of these diseases, with Alzheimer’s disease (AD) being the most common form. The diagnosis of neurodegenerative disorders, and especially the different dementia forms, is cumbersome due to the large overlap of clinical symptoms^4,5^. Consequently, the need for specific and reliable biomarkers is growing, not only to identify the disease, but also for monitoring the disease progress and to evaluate the efficiency of new potential treatments. Moreover, biomarker research can help identify proteins potentially involved in disease pathology which, through follow-up studies, may reveal novel pathways linked to the disease and its underlying mechanisms. Ultimately, this can also lead to the discovery of new therapeutic targets.

A cost effective and relatively non-invasive approach to monitor pathological processes involves measuring protein levels in biofluid samples. Although blood-derived samples are less invasive, cerebrospinal fluid is considered a more informative sample type due to its close proximity to the brain tissues^6^. Amyloid beta (Aβ) 1–42 peptide and phosphorylated tau (p-tau) are cerebrospinal fluid (CSF) biomarker indicative of the presence of Aβ plaques and tau-neurofibrillary tangles in the brain tissue, the hallmarks of AD. These biomarkers are now broadly used to aid in AD diagnosis as well as to select participants for clinical studies^7^.

Although the number of protein biomarker studies has been steeply increasing in recent years, only a handful of biomarkers have been implemented in clinical practise, partially due to the challenges associated with biomarker research and validation^8^. One of these challenges is the presence of inter-individual variability in CSF protein levels unrelated to disease, which can lead to low biomarker performance^9,10^. Although the sources of such variability are complex, indirect adjustments can be made to reduce its impact. A good example used in practice is the CSF Aβ42 in ratio with Aβ40, where Aβ40 works as an adjustment factor, increasing the accuracy of Aβ42 as a predictor for the presence of amyloid plaques in the brain^11,12^. Interestingly, several studies, including ours, revealed relatively strong correlations between Aβ40 and p-tau^13,14^ as well as between other CSF brain-derived proteins across various neurodegenerative diseases and in healthy individuals^13,15–21^. This broad co-variance pattern among CSF proteins may indicate possible differences in overall levels of brain-derived proteins between individuals, which might be driving the primary variance in CSF protein levels independently of pathology.

We have previously demonstrated that adjusting for the overall levels of brain-derived proteins through proteins ratios enhanced the performance of biomarkers in a clinical AD cohort^21^. Building upon this work, we here expand our research to a spectrum of neurodegenerative diseases including AD, amyotrophic lateral sclerosis (ALS), behavioural variant frontotemporal dementia (bvFTD), corticobasal syndrome (CBS), primary progressive aphasia (PPA), and progressive supranuclear palsy (PSP). Analysing a panel of 69 proteins in CSF, pre-selected based on previous in-house studies^13,15,22–26^ and literature, we initially explore the overall variability in the dataset and reveal the presence of inter-individual variability in general levels of brain-derived CSF proteins. Subsequently, we evaluate different approaches to adjust for this variability in order to improve biomarker performance. Importantly, we compare the observed CSF profiles across the included diseases to discern differences and similarities, providing valuable insights into disease-specific and unspecific protein signatures.

## Methods

### Participants

The 499 participants included patients with Alzheimer´s disease (AD) (n = 69), primary progessive aphasias (PPA) (n = 166), behavioural frontotemporal demenia (bvFTD) (n = 129), corticobasal syndrome (CBS) (n = 26), progressive supranuclear palsy (PSP) (n = 39), amyotrophic lateral sclerosis (ALS) (n = 35), and healthy controls (n = 35) (Table 1). Diagnosis was based on internationally established criteria. Participants were examined between 05/2011 and 07/2020 within the FTLD (frontotemporal lobar degeneration) Consortium study^27,28^. Each participant passed through comprehensive clinical and neuropsychological tests, as well as CSF and blood sampling and magnetic resonance imaging (MRI). CSF was obtained by lumbar puncture. The FTLD Consortium study applies strict standard operating procedures (SOP) to guarantee data reliability. The study was conducted following the Declaration of Helsinki and approved by the local ethics committees of all centres involved (University of Ulm #39/11). An additional ethical approval for the presented study was received from the Swedish Ethical Review Authority (2022-02653-01). All participants in both cohorts gave written informed consent for sample and data collection.

**Table 1:** Cohort description.

	AD	ALS	bvFTD	CBS	PPA	PSP	Healthy control
N	69	35	129	26	166	39	35
Female [N](%)	40 (58%)	15 (43%)	48 (37%)	14 (54%)	77 (46%)	20 (51%)	19 (54%)
Age^a^	70 (47–83)	66 (42–85)	62 (41–82)	69 (54–82)	68 (44–86)	68 (50–85)	73 (50–89)
Aβ42^a^ [pg/ml]	498 (202–1415)	897 (393–1828)	784 (251–1950)	736 (252–1461)	776 (210–2087)	661 (318–1445)	–
Aβ40^a^ [pg/ml]	9789 (783–20 010)	10 516 (4627–20 301)	9112 (2756–20 158)	9710 (3000–13 221)	10 780 (2948–21 447)	8719 (3787–17 438)	–
Aβ42/40^a^	0.046 (0.029–0.185)	0.093 (0.029–0.118)	0.095 (0.036–0.122)	0.086 (0.045–0.116)	0.091 (0.024–0.123)	0.093 (0.030–0.116)	–
t-tau^a^ [pg/ml]	638 (170–2000)	376 (145–953)	334 (88–2,000)	354 (98–1109)	402 (82–2,000)	284 (110–1,672)	–
p-tau^a^ [pg/ml]	106 (12–352)	36 (15–134)	40 (10–378)	46 (14–164)	48 (10–381)	40 (16–253)	–
Q-alb^b^	6.09 (2.80–14.60)	6.40 (2.80–18.20)	6.10 (2.60–23.50)	5.37 (2.70–17.00)	6.20 (2.70–14.20)	5.80 (2.90–24.60)	–
CDR score^a,c^	5.2 (0.5–14.0)	2.8 (0.0–17.0)	5.0 (0.0–17.0)	4.0 (0.0–11.0)	2.5 (0.0–16.0)	3.8 (0.0–18.0)	–

^a^Data is presented in the format: median (range).

^b^Q-Alb – missing data [N]: AD: 19 (28%), ALS: 4 (11%), bvFTD: 29 (22%), CBS: 3 (12%), PPA: 53 (32%), PSP: 8 (21%).

^c^CDR score – missing data [N]: AD: 11 (16%), ALS: 7 (20%), bvFTD: 17 (13%), CBS: 6 (23%), PPA: 27 (16%), PSP: 9 (23%).

### CSF amyloid and tau measurements

Quantification of CSF Aβ40, Aβ42 and p-tau181 and total tau (t-tau) was conducted using the chemiluminescent enzyme immunoassay on the on the automated Lumipulse G600II analyzer according to the instructions (Fujirebio). Albumin in CSF and serum was measured by standard immunochemical nephelometry^29,30^. To analyze the association between CSF AD markers and median brain-derived protein levels, individuals were classified based on their CSF amyloid and tau status, independent of diagnosis^31^. Individuals with Aβ42/40 ≤ 0.07 were classified as amyloid positive (A +) and with p-tau ≥ 60 [pg/ml] as tau positive (T +), resulting into four groups, A-T- (n = 258), A + T- (n = 30), A + T + (n = 142) and A-T + (n = 34) (Supp. Figure 1). Only patient samples are included in these groups, as amyloid and tau levels were not routinely measured for healthy individuals, and were therefore not available in this study.

### CSF protein analysis using suspension bead array

The selection of proteins to include in this study was mainly based on prior in-house studies^13,15,22–26^. The pre-selected proteins were analysed using a multiplex antibody-based suspension bead array employing polyclonal antibodies produced in rabbits within the Human Protein Atlas project (www.proteinatlas.org). Each antibody targeting a selected protein was attached to the surface of color-coded magnetic beads (MagPlex, Luminex corp.) through NHS-EDC (N-Hydroxysuccinimide and 1-Ethyl-3-(3-dimethylaminopropyl)carbodiimide) chemistry as previously described by Pin et al.^32^. The antibody-coupled beads were pooled into two separate bead arrays, each containing bead-IDs corresponding to the intended protein target analysis at different sample dilutions (either 1/25 or 1/200) (Supp. Table 1).

The CSF samples were distributed into 96-well PCR plates in a stratified randomisation manner based on diagnosis, age and sex of the individuals as well as the sample collection site. The crude samples were then directly labelled with biotin (NHS-PEG4-biotin, A39259, ThermoFisher Scientific) at an approximate tenfold molar excess, following established procedures^32^. The biotin-labelled samples were diluted with a buffer containing bovine serum albumin (BSA) and rabbit IgG to a final dilution of 1/25 and 1/200. The diluted samples were further heat-treated for 30 min at 56 °C before incubation with the prepared bead arrays at room temperature overnight. Following the washing of unbound proteins, the antibody-bound protein targets were incubated with a streptavidin-coupled fluorophore. The signal read-out was facilitated using a Flexmap 3D instrument (Luminex corp.). The data per bead ID (protein target) and sample was acquired as a median fluorescent intensity, yielding relative protein quantities. The obtained data were further adjusted for delayed instrument readout using robust linear model as described previously^21^, and for sample plate batch-effect using removebatchEffect (limma). To evaluate the intra-assay variability of each protein assay, three technical replicates, composed of 122 pooled CSF samples from individuals with neurodegenerative disease, were included in each 96-well sample plate. Further, one 96-well sample plate was re-analyzed to evaluate inter-assay correlation. Data analysis was conducted on 69 proteins passing the quality control (Supp. Table 1), with the median inter-assay Spearman’s correlation of 0.95 (range 0.84–0.99) and median intra-assay coefficient of variation (CV) of 4.3% (range 2.3– 9.9%).

### Data analysis and visualizations

The open-source R statistical software (version 4.2.2) was used for all data processing, analysis, and visualization, incorporating supplementary packages tidyverse, tidymodels, ggpubr, ggbeeswarm, ggrepel, pheatmap, stats, scales, and patchwork. Details of additional packages and functions employed in specific data analysis sections are provided therein. To enhance the figure clarity and readability (e.g., to adjust figure legends) we performed additional refinements using the vector graphic editor Affinity Designer (version 1.8.6) by Serif, West Bridgford, UK.

For all analysis, relative protein levels measured using the suspension bead array were log2 transformed and centred to median (per protein). Correlation between the measured proteins was calculated on the full sample cohort using Spearman’s correlation (cor, stats), and protein clustering based on correlation was performed using the average linking method. Whether proteins were classified as brain-elevated was determined based on the tissue transcriptomic data classification of genes from the Human Protein Atlas^33^. Principal component analysis was performed on log2 transformed data centred to mean and scaled to standard deviation. The median brain-derived protein levels were determined by calculating the median level of cluster 1 proteins per sample. The differences in the median brain-derived protein levels between males and females were evaluated using the Wilcoxon’s two-sided test, and between the diagnostic groups using the Kruskal–Wallis test followed by Dunn’s test for pairwise comparisons, with Benjamini–Hochberg adjustment for multiple testing^34^. Adjusted p-values < 0.05 were considered significant.

#### Linear regression models

Generalized linear models (glm, stats) were used to evaluate whether adjustment for overall levels of brain-derived proteins estimated by the sample median of cluster 1 proteins increased association of the analysed CSF proteins to individual diseases/healthy state. For this, two types of models were constructed for each measured protein and each comparison of two different diagnosis. In each model, the measured CSF protein levels were used as an outcome variable. In the first model, diagnosis was used as the main predictor and in the second model both diagnosis and median brain-derived protein levels were used as predictors. Both models were further adjusted for sex and age. To compare the association of CSF protein levels of the analysed protein to the tested diagnosis with and without adjustment to general protein levels, the beta coefficient for diagnosis along with the p-value were extracted from the model. The extracted p-value was further adjusted for multiple comparisons using the Benjamini–Hochberg method. Adjusted p-values < 0.05 were considered significant.

#### Logistic regression models

To predict diagnosis, logistic regression models (glm, stats) were constructed. Two types of diagnosis predictions were investigated in this study, distinguishing individual diseases from healthy controls and distinguishing individual diseases from all other diseases. For both cases, several different predictor combinations were tested: (1) only median brain-derived protein levels, (2) only individual CSF protein levels, (3) cluster 1 protein sample median + individual CSF proteins, (4) CSF protein pair combinations. Given the limited size of the sample groups, model performance was evaluated using cross-validation, with the area under the receiver operating characteristic (ROC) curve (AUC) as the primary metrics. Further, to address imbalances in sample group sizes, undersampling to the size of the smaller group was applied. Each model iteration involved 15 rounds of undersampling with 3 partitions and 5 repeats in cross-validation for each undersampling round. The resulting AUC represented the median AUC across the 15 undersampling rounds, reported with the corresponding interquartile range (IQR) in the example ROC-curve plots and tables. The ROC-curves for example models were constructed using average sample predictions from the undersampling round resulting in the median AUC.

## Results

### Median CSF levels of co-varying brain-derived proteins represent the main variability between individuals

**Fig. 1:**
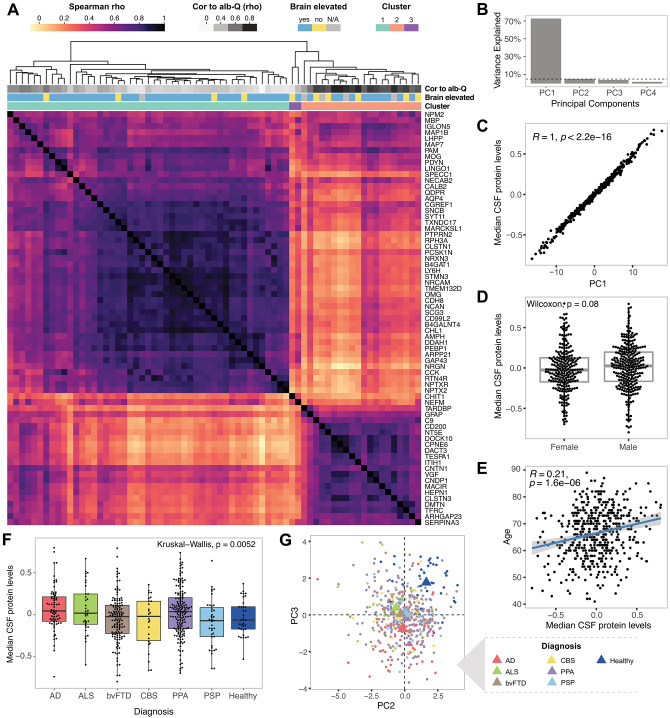
Median CSF levels of co-varying brain-derived proteins reflect the main variability between individuals. (**A**) A heatmap showing Spearman correlations between the measured proteins using the full sample cohort of 499 individuals. The heatmap is annotated with correlation of the individual proteins to albumin CSF/serum quotient, and classification of the proteins based on specificity to brain tissues according to the transcriptomic data provided in the Human Protein Atlas (www.proteinatlas.org). (**B**) Variance in the data explained by the first four principal components from principal component analysis (PCA) performed on all samples using cluster 1 proteins. (**C**) Pearson correlation between PC1 and median levels of cluster 1 proteins per individual. (**D**) Comparison of median levels of cluster 1 proteins in males and females in the full cohort. (**E**) Pearson correlation between age and median levels of cluster 1 proteins. (**F**) Comparison of median levels of cluster 1 proteins between individuals stratified based on diagnosis. (**G**) Visualization of separation of healthy individuals from the rest of the cohort provided by PC2 and PC3.

Based on protein-to-protein correlations within the full cohort of 499 individuals, the 69 measured CSF proteins clustered in three main groups (Fig. 1A). Cluster 1 predominantly comprised proteins of brain origin, with weak correlation with the albumin CSF/serum quotient. The second cluster (cluster 2) instead comprised proteins with stronger correlation to albumin quotient, suggesting either their transfer to CSF from blood^35^, or low detectability of these proteins in CSF. Both complement component 9 (C9) and inter-alpha-trypsin inhibitor heavy chain 1 (ITIH1), which were included as controls for blood-derived proteins, clustered to this group. Two proteins, neurofilament medium (NEFM) and Chitinase 1 (CHIT1) clustered separately (cluster 3) as they did not correlate well to proteins in the two other clusters. Principal component analysis (PCA) performed on the highly-intercorrelated proteins included in the predominantly brain-derived cluster 1 showed that over 70% of the variability in the data could be explained by median protein levels per sample, calculated from the proteins included in this cluster (Fig. 1B,C). That is, some individuals in the cohort had consistently low levels across these proteins while other individuals had consistently high levels. Next, we investigated whether the differences in median levels of proteins in cluster 1 could be associated with age, sex or diagnosis. While no significant differences were observed between the sexes (Fig. 1D), age demonstrated weak, yet statistically significant, positive correlation (Fig. 1E). Subtle differences were also observed between the individual diagnostic groups (Fig. 1F), however there was substantial overlap in the observed variance among the groups and only bvFTD compared to AD demonstrated a statistically significant difference (Supp. Table 2). This suggests that factors other than sex, age or diagnosis primarily contributed to the variance of these proteins among individuals. Notably, while PC1 (~ median protein levels of cluster 1 proteins) did not show large variance between the studied diagnostic groups, PC2 and PC3 partially separated healthy individuals from those with neurodegenerative diseases (Fig. 1G). However, PC2 and PC3 together only accounted for ~ 10% of the observed variance in the data (Fig. 1B). We hypothesised that adjusting the measured protein data for median brain-derived protein levels could decrease the influence of inter-individual variability in general protein levels, and potentially improve the diagnostic performance of the proteins.

### Adjusting for median brain-derived protein levels increases the performance of proteins in disease vs. healthy classification

**Fig. 2:**
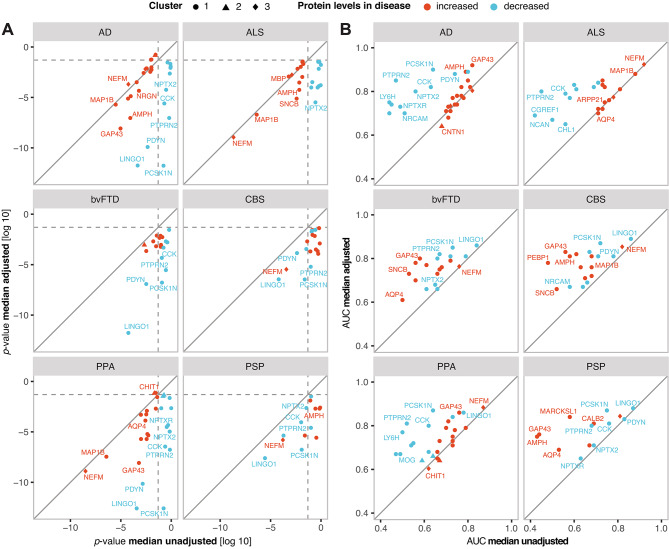
Association with disease and predictive accuracy of individual CSF proteins increases when adjusted for median CSF protein levels. (**A**) A linear model was constructed for each protein and disease, where the protein levels were the outcome variable. In the figure, the p-values for the diagnosis predictor (each disease vs. healthy control) are compared for models adjusted (y-axis) and unadjusted (x-axis) for median CSF protein levels. Only proteins with significant p-value in at least one of the models are included in the figure. The dashed line is drawn at p-value = 0.05. (**B**) ROC-AUCs resulting from logistic regression models predicting diagnosis (each disease vs. healthy controls) using the adjusted protein levels (y-axis) compared to unadjusted protein levels (x-axis). Only proteins with significant p-value in at least one of the models in (**A**) are included.

To test whether adjustment for median brain-derived protein levels could enhance the association of measured proteins with disease, linear regression models were constructed for each protein and individual disease. We then compared p-values for diagnosis (in comparison to healthy controls) with and without adjustment for the median brain-derived protein levels, while controlling for age and sex in all models. As presented in Fig. 2A, the adjustment increased association with individual diseases for the majority of the measured proteins showing significant alteration in disease prior to adjustment. Moreover, several additional proteins, particularly those decreased in the studied diseases, reached statistically significant results only after the adjustment. Next, we compared the predictive accuracy for disease vs. healthy controls adjusted and unadjusted individual proteins using logistic regression models, with AUC as the selected metrics. As anticipated, we observed improved performance for the majority of the proteins when adjusted for the median CSF protein levels (Fig. 2B).

### The majority of altered proteins are not disease specific

**Fig. 3:**
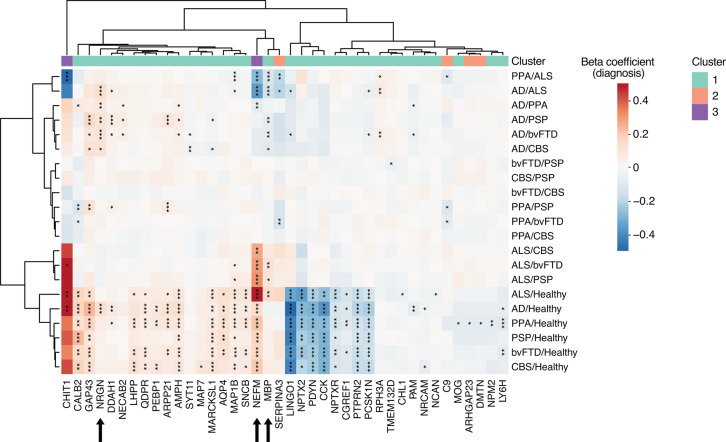
Protein significantly altered in individual diseases compared to healthy controls and/or between the diseases. Heatmap constructed using the beta coefficients for diagnosis from linear models adjusted for median CSF protein levels. Beta coefficients exceeding an absolute value of 0.5 were constrained to 0.5 for visualization purposes. The stars indicate the significance level measured with the p-value: *< 0.05; **< 0.01, ***< 0.001. Only proteins with significant difference in at least one of the comparisons are included. The proteins are annotated with the clustering results based on protein correlation (Fig. 1A).

We further investigated the differences in protein levels across the studied diseases. For this, we constructed linear models for comparison between each disease pair, with adjustment median brain-derived protein levels, sex and age. As presented in Fig. 3, twenty proteins showed significant differences in CSF levels between the diseases for at least one comparison. Among these proteins, the majority was increased in AD compared to other diseases, with neurogranin (NRGN) showing the highest specificity for AD. Significant differences were also observed for myelin basic protein (MBP) and neurofilament medium (NEFM), which both showed increased levels in ALS compared to other diseases. However, it is important to note that NEFM also demonstrated higher levels in all diseases compared to healthy controls, indicating a general increase in all the diseases but with larger difference in ALS. Similar results were also obtained from the logistic regression models evaluating the predictability of each measured protein (adjusted for median brain-derived protein levels) for individual disease against all the other diseases. Based on these outcomes, the majority of the proteins showed low disease specificity (Supp. Fig. 2), with only NEFM, NRGN and MBP reaching AUC > 0.7 for one of the diseases versus the others at the same time as for the disease versus healthy controls (Table 2).

**Table 2:** The three proteins with AUC > 0.7 for classification of the respective disease vs other diseases as well as disease vs healthy.

Diagnosis	Protein	AUC (IQR) from other diseases	AUC (IQR) from healthy controls
AD	NRGN	0.73 (0.72–0.74)	0.82 (0.80–0.84)
ALS	NEFM	0.77 (0.75–0.79)	0.92 (0.91–0.92)
ALS	MBP	0.72 (0.70–0.77)	0.81 (0.80–0.81)

### Protein pairs adjust for median brain-derived protein levels and enhance classification performance

**Fig. 4:**
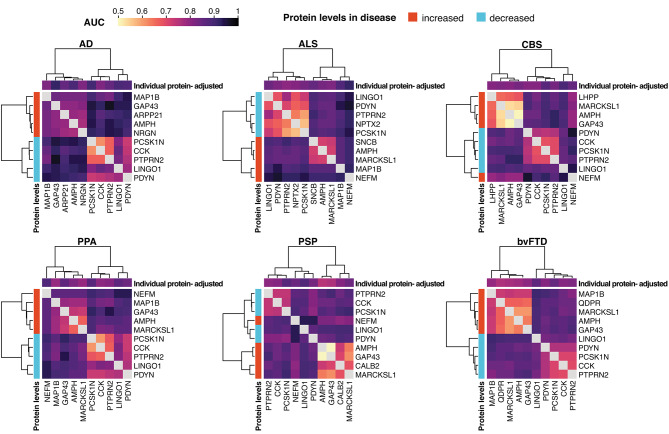
Diagnostic accuracy provided by paired CSF proteins. Heatmaps constructed with AUC values from logistic regression models predicting diagnosis (each disease vs. healthy control) using protein pairs. For each disease, the selected top five increased and top five decreased proteins (adjusted levels) with the best individual performance in separating the affected individuals from healthy controls were included in the models. Each heatmap is annotated on the left with the direction of the alteration in disease (increased/decreased), and on top with AUC values from logistic models predicting diagnosis (individual disease vs. healthy control) using single proteins adjusted for median CSF levels. AUC values < 0.5 are replaced with the value of 0.5.

While adjusting for median brain-derived protein levels enhanced the performance of CSF proteins, it may not be a practical approach. This is particularly true for research studies or in clinical settings where only one or a limited number of proteins is measured, making the estimation of the median brain-derived protein levels unfeasible. Instead, correlated proteins can be used in pairs to adjust for inter-individual variability. Moreover, proteins in the pair with opposite alteration in disease may further increase the diagnostic performance. To test this, we selected the top five elevated and decreased proteins per disease with the best performance in separating the affected individuals from healthy controls when adjusted for median brain-derived protein levels (Supp. Table 3, Supp. Fig. 3). All protein pair combinations within the 10 selected proteins per disease where then evaluated using logistic models, with the AUC as the evaluation metrics. For all the six investigated diseases, the protein pair combinations with one protein elevated and one decreased in the investigated disease showed the best performance, which was either comparable or higher compared to the performance of the individual proteins adjusted for median brain-derived protein levels (Fig. 4, Supp. Figs. 4, 5). Furthermore, in ALS, a high performance was observed for all protein combinations including NEFM, suggesting that in this case NEFM is the main contributor to the separation.

**Fig. 5:**
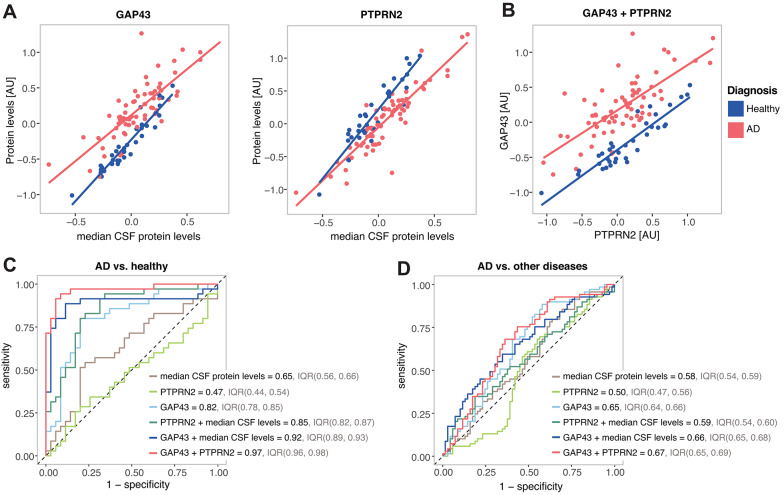
Relationship between GAP43 and PTPRN2 and their diagnostic performance. Examples for AD and healthy controls: (**A**) Scatterplots illustrating the relationship between median CSF protein levels and GAP43 and PTPRN2 levels respectively. (**B**) Scatterplot illustrating the relationship between GAP43 and PTPRN2 levels. (**C**,**D**) Examples of ROC-curves showing the predictive performance for AD vs. Healthy controls (**C**), and for AD vs. other diseases (**D**), using median CSF protein levels, GAP43 and PTPRN2 levels individually, and their combinations.

To better illustrate the adjustment and the additive effect of the protein pairs, we selected growth associated protein 43 (GAP43) with protein tyrosine phosphatase receptor type N2 (PTPRN2), which provided the best result in separating AD from healthy controls, as an example. Figure 5A shows the separation of AD individuals from healthy controls provided by GAP43 and PTPRN2 individually, when adjusted for median brain-derived protein levels. Figure 5B further shows the separation provided by GAP43 and PTPRN2 combined. Presented altogether, the performance of the individual and combined components for the AD vs. healthy controls classification using median CSF protein levels, GAP43 and PTPRN2 is compared in Fig. 5C. The median CSF protein levels, PTPRN2 and GAP43 alone showed the lowest performance (AUC = 0.65, 0.47 and 0.82, respectively). In comparison, PTPRN2 and GAP43 individually but adjusted for median brain-derived protein levels showed considerably increased performance (AUC = 0.85 and 0.92, respectively). Lastly, GAP3 with PTPRN2 in pair demonstrated the best performance with AUC = 0.97. However, the pair was not able to separate AD from the other diseases (AUC = 0.67), although its performance was better compared to the alternatives (Fig. 5D).

### CSF amyloid and tau correlate with median brain-derived protein levels

**Fig. 6:**
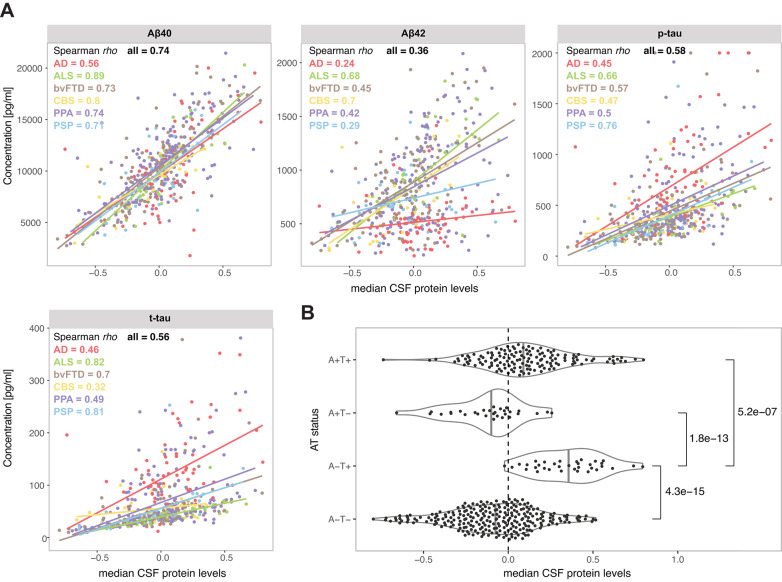
Relationship between median CSF protein levels and CSF concentrations of amyloid and tau. (**A**) Scatterplots illustrating the relationship between Aβ40, Aβ42, p-tau and t-tau and median CSF protein levels for all individuals as well as for individual diseases. (**B**) Distribution of median CSF protein levels in groups of participants divided by amyloid and tau (AT) status determined with CSF Aβ42/Aβ40 and p-tau levels.

Many of the proteins investigated in this study have been previously shown to correlate with the established AD CSF amyloid and tau markers^13,15–20^, suggesting that the established markers may also be affected by the inter-individual variability in general CSF protein levels. All samples in this cohort, except healthy controls, were analysed for Aβ40, Aβ42, p-tau and t-tau (Table 1). All four markers showed correlation with median brain-derived protein levels in this cohort (Fig. 6A). Notably, the strongest correlation was observed with Aβ40 (rho = 0.74 for all samples). Contrary to Aβ42, CSF p-tau and t-tau are not commonly adjusted with Aβ40 or other protein levels. Therefore, individuals with not only high CSF tau levels but also high general CSF protein levels could be falsely denotated as CSF-tau positive based on the set clinical cut-offs. To explore whether this could be the case, we compared the distribution of the median brain-derived protein levels in sample group categorised based on the CSF Aβ42/40 and p-tau status (Supp. Table 4). As anticipated, the amyloid-negative but tau-positive individuals (A-T +) showed generally higher median brain-derived protein levels compared to all the other groups (Fig. 6B), indicating a possible false p-tau-positivity derived from overall high CSF protein levels in this group.

## Discussion

The utility of a biomarker often relies on an alteration in its levels from a healthy state. However, this change might be relatively small compared to the naturally occurring variability among individuals, which makes the detection of pathology-related protein alterations challenging. In this study, we show that CSF levels of brain-derived proteins co-vary with their median level (here referred to as median brain-derived protein levels), which represent the main source of variability in the brain-derived proteins between individuals. We further demonstrate that adjusting for the median levels increases the association of the individual proteins with disease as well as improve their performance in distinguishing individuals with disease from healthy controls. In addition, we show that this adjustment can be simplified by combining proteins in pairs, where one of the proteins increases and one decreases in the studied disease. Using this approach, we achieved adjustment for individual-dependent overall protein levels and further increased the classification performance through the additive effect of the two proteins with opposite alteration.

Based on the data in this cohort of 499 individuals, the median CSF levels of brain-derived proteins did not significantly differ between males and females. However, weak but significant positive correlation was detected between the CSF median levels and age in this cohort. This is in concordance with previous studies indicating higher levels of many CSF proteins in older individuals, possibly due to decreased CSF turnover in older age^36–38^. We also observed significant differences in median CSF protein levels between the diagnostic groups, with slightly higher levels in AD, ALS and PPA. This subtle difference may stem from the limited protein selection, wherein many proteins exhibit more pronounced increases in these diseases, consequently slightly elevating the overall group median. Alternatively, variations in CSF physiology across the groups could contribute to the observed differences^39^. Despite this, the differences were modest, with substantial overlap in variance between the groups. This suggests that inter-individual variability in median CSF levels of brain-derived proteins is not primarily driven by disease pathology. Instead, physiological factors such as differences in CSF brain volume, CSF production rate and/or clearance may contribute to the differences in median CSF levels of brain-derived proteins observed among individuals. However, these factors were not examined in this study, and further research is needed to identify the primary contributors to this variability.

Consistent with our hypothesis that inter-individual variability in general levels of brain-derived proteins partially masks the variability driven by pathological changes, we found that majority of the measured proteins with significantly altered levels in the disease groups strengthened their association with the disease after adjustments for median brain-derived protein levels. Our findings are also in line with previous work by Opsahl et al.^40^, who proposed a normalization approach based on brain-derived proteins to reduce the influence of non-CNS proteins, such as IgGs, that can skew global normalization outcomes. They demonstrated that normalizing CSF protein abundances specifically to CNS-derived proteins resulted in significantly reduced intergroup variability compared to more traditional methods, such as normalization to mean or median overall protein intensities. Parallel to our study, Karlsson et al.^41^ also discovered that highly abundant CSF proteins, which are likely to be studied in the context of brain diseases, covary with the mean standardized CSF protein levels and further demonstrated that adjustment for this covariance using reference proteins increased the accuracy of established CSF biomarkers for amyloid and tau AD pathology. Together, these and our findings emphasize the importance of addressing non-pathological variability to improve performance of both existing as well as new potential CSF biomarkers for brain diseases.

Here, we further focused on multi-disease comparison of the observed patterns. We found several proteins consistently altered across all the diseases. Leucine rich repeat and Ig domain containing 1 protein (LINGO1), neuronal pentraxin 2 (NPTX2), prodynorphin (PDYN), cholecystokinin (CCK), neuronal pentraxin receptor (NPTXR), PTPRN2 and proprotein convertase subtilisin/kexin type 1 inhibitor (PCSK1N) were found significantly decreased in all six diseases compared to healthy controls. Previous studies have also reported reductions in these proteins in CSF across various neurodegenerative disorders^26,42–50^. Importantly, in our study cohort, most of these alterations reached statistical significance only after adjustment for median brain-derived protein levels. Conversely, NEFM, GAP43, amphiphysin (AMPH), myristoylated alanine-rich C-kinase substrate like 1 (MARCKSL1) and calbindin 2 (CALB2) showed consistent increase in all six diseases. Among these, NEFM, GAP43 and AMPH have previously been identified as elevated in CSF in association with neurodegeneration^15,19,23,26,51,52^. Like GAP43, MARCKSL1 is a substrate of protein kinase C and is suggested to play a role in the regulation of pre- and post-synapse morphologies^53,54^. The increase in CSF levels of MARCKSL1 and GAP43 may thus be related. CALB2, a calcium-binding protein highly expressed in neurons, has previously been found elevated in CSF from individuals with Niemann-Pick disease, along with NEFL and tau^55–57^, suggesting a potential association to neuronal damage. The considerable overlap in altered proteins across the studied diseases indicates that changes in the levels of many CSF proteins may stem from common mechanisms to the diseases, potentially reflecting general neurodegenerative processes and/or neuronal loss. While such biomarkers may offer limited utility for differential diagnostics, they hold promise as valuable tools for monitoring disease progression and assessing treatment efficacy.

When comparing CSF protein patterns across the six diseases, only three proteins exhibited some degree of specificity toward a particular disease when adjusted for median brain-derived protein levels. NRGN was found to be elevated in AD compared to all other diseases and healthy controls. This observation aligns with previous cross-disease investigations conducted by Wellington et al.^58^ and Portelius et al.^59^, where NRGN levels were elevated in AD but not in FTD, Lewy body dementia, Parkinson’s disease, multiple system atrophy, PSP, PPA, ALS, or CBS. Further, we observed MBP to be significantly increased in ALS compared to the other diseases, except CBS (potentially due to low number of individuals in this group), as well as compared to healthy controls. MBP, an abundant component of the myelin membrane, is considered to be a nonspecific marker of CNS inflammation^60^, and has previously been studied as a potential biomarker for multiple sclerosis, showing elevated CSF levels in the disease^61,62^. Recently, Yue Li et al*.* demonstrated an increase in CSF MBP in 16% of patients with ALS, with a higher increase in those with comorbid bvFTD^63^. In our study, we did not observe any significance difference between the included ALS subgroups (ALS, ALS + bvFTD, ALS + PPA, data not shown). However, it is important to note that both studies had limited participant numbers, thus further research is needed to determine the significance of MBP as biomarker in the context of ALS. Lastly, NEFM showed significant elevation in ALS compared to the other diseases. However, NEFM was also significantly elevated in all the diseases compared to other controls, as discussed earlier, indicating a non-specific increase in neurodegenerative conditions, with the most pronounced increase in ALS. This pattern aligns with the observations for NEFL^64^, suggesting that both reflect similar processes, although NEFM is not as extensively studied.

We further suggest an alternative method for addressing inter-individual variability in general CSF levels of brain-derived proteins by utilizing proteins in pairs. This approach is particularly valuable for implementing biomarkers in clinical practice, where precise and reliable procedures are essential, and the number of measured proteins is limited. Furthermore, it allows for further increase in predictive accuracy through additive effect when correlated proteins with opposite alterations in CSF are combined. Building upon our previous work focused on AD^21^, we demonstrate here the applicability of this adjustment method in the broader context of neurodegenerative diseases. The use of biomarker ratios is not a novel concept. For instance, commonly employed protein ratio in both research and in clinical practice is that of CSF Aβ42 to Aβ40, a method used to determine the presence of amyloid pathology. In this context, Aβ40 is considered an adjustment factor correcting for the individual-specific variability in amyloid production levels^12^. Here we show that Aβ40 strongly correlates with median brain-derived protein levels, indicating that Aβ40 may work not only as an adjustment factor for amyloid production but also for the overall CSF protein levels derived from the individual-specific CSF dynamics. Notably, we also observed a moderately strong correlation between median brain-derived protein levels and p-tau and t-tau, suggesting that these markers are likely also affected by the interindividual variability in the overall CSF protein levels and could thus benefit from adjustment. This hypothesis is supported by previous work reported by Guo et al*.* who demonstrated that CSF adjusting p-tau181 with Aβ40 enhances predictive accuracy for the presence of tauopathy compared to p-tau181 alone^65^. Overall high CSF concentration of brain-derived proteins could also explain the existence of a clinically controversial group of individuals with amyloid-negative but tau-positive status as determined based on CSF Aβ42/Aβ40 and p-tau. According to our data, individuals in this group have considerably higher median brain-derived protein levels compared to the other diagnostic groups, suggesting that elevated tau levels in this group may not necessarily denote tau pathology but rather be a result of impaired CSF dynamics causing overall elevation in CSF protein concentrations, as also suggested by Eriksson et al.^66^ and Karlsson et al.^41^.

This study has several limitations that require consideration. One constraint is the relatively small number of measured proteins, which are pre-selected and may not fully represent the CSF protein repertoire. This partial representation could potentially impact the accuracy of the determined median CSF levels of co-varying brain-derived proteins. The presented study focuses on normalization strategies for the co-varying brain-derived proportion of proteins in CSF; however, different approaches may be needed for addressing disease-unrelated variations in levels of other brain-derived proteins or proteins originating from plasma or having a mixed CNS/peripheral origin. Due to the limited number of healthy controls, the protein clustering is performed based on correlations between the proteins constructed from the full sample cohort. This might lead to weaker correlations for proteins that alter with pathology. Further limiting is the lack of direct validation in an independent cohort, which was not available for this study. Although our findings are well supported by previous research, validation in larger multi-disease cohorts would strengthen our conclusions. Additionally, it would be beneficial to investigate protein levels in relation to genetic variants, which may contribute to protein variability between individuals. Given the cross-sectional nature of this study, the identified proteins and protein pairs cannot be evaluated as prospective biomarkers. To assess their potential for monitoring disease progression, further investigation using longitudinal samples and clinical follow-up is necessary. Finally, it would be valuable to compare the adjusted protein data with other types of biomarkers such as MRI and/or PET images to determine their concordance.

## Conclusion

In conclusion, our study highlights the impact of inter-individual variability in cerebrospinal fluid (CSF) protein levels on the diagnostic utility of biomarkers for neurodegenerative diseases. We demonstrate that adjusting for median CSF protein levels of co-varying brain-derived proteins significantly enhances the association of these proteins with disease and improves their diagnostic performance. A simplified adjustment method using pairs of proteins with opposing alterations further increases biomarker accuracy, which could be valuable for clinical applications. Our findings also reveal that many CSF protein alterations are shared across multiple neurodegenerative conditions, suggesting a common underlying mechanism that may limit their specificity but provide insight into general neurodegenerative processes. Further studies of independent cohorts are needed to establish which pairs are most informative and specific for individual diseases. Additionally, given our findings suggesting that overall CSF levels of brain-derived proteins are not primarily driven by pathology, we propose that such adjustments could benefit CSF across other CNS-associated fields, including neuroinflammatory and psychiatric biomarker studies.

## Supplementary Information

Supplementary Information.

## Data Availability

The datasets used and/or analysed during the current study are available from the corresponding author on reasonable request.

## Abbreviations

AD: Alzheimer’s disease
ALS: Amyotrophic lateral sclerosis
AMPH: Amphiphysin
AUC: Area under the curve
Aβ40: Amyloid beta 40 residues long
Aβ42: Amyloid beta 42 residues long
bvFTD: Behavioural variant frontotemporal dementia
CALB2: Calbindin 2
CBS: Corticobasal syndrome
CCK: Cholecystokinin
CSF: Cerebrospinal fluid
CV: Coefficient of variation
FTLD: Frontotemporal lobar degeneration
GAP43: Growth associated protein 43
LINGO1: Leucine rich repeat and Ig domain containing 1
MARCKSL1: Myristoylated alanine-rich C-kinase substrate like 1
MBP: Myelin basic protein
MND: Motor neurone disease
NEFL: Neurofilament light chain
NEFM: Neurofilament medium chain
NHS: N-Hydroxysuccinimide
EDC: 1-Ethyl-3-(3-dimethylaminopropyl)carbodiimide
NPTX2: Neuronal pentraxin 2
NPTXR: Neuronal pentraxin receptor
NRGN: Neurogranin
p-tau: Phosphorylated tau
PCA: Principal component analysis
PCSK1N: Proprotein convertase subtilisin/kexin type 1 inhibitor
PDYN: Prodynorphin
PEG: Polyethylene glycol
PPA: Primary progressive aphasia
PSP: Progressive supranuclear palsy
PTPRN2: Protein tyrosine phosphatase receptor type N2
Q-Alb: Albumin CSF/serum quotient
ROC: Receiver operating characteristic
t-tau: Total tau
