# Supplementary material for: Addressing inter individual variability in CSF levels of brain derived proteins across neurodegenerative diseases
Source: Sci Rep. 2025 Jan 3;15:668. doi: 10.1038/s41598-024-83281-y (PMC11698900; doi:10.1038/s41598-024-83281-y)
Supplement: Supplementary file 1 — Supplementary Information. [file 41598_2024_83281_MOESM1_ESM.pdf]

SUPPLEMENTARY MATERIAL

Addressing inter individual variability in CSF levels of brain derived proteins across neurodegenerative diseases

Sára Mravinacová[1], Sofia Bergström[1], Jennie Olofsson[1], Nerea Gómez de San José[2], Sarah Anderl-Straub[2], Janine Diehl-Schmid[3,4], Klaus Fassbender[5], Klaus Fliessbach[6], Holger Jahn[7], Johannes Kornhuber[8], G. Bernhard Landwehrmeyer[2], Martin Lauer[9], Johannes Levin[10,11,12], Albert C. Ludolph[2,13], Johannes Prudlo[14], Anja Schneider[6], Matthias L. Schroeter[15], Jens Wiltfang[16,17], Petra Steinacker[18], FTLD consortium, Markus Otto[2,18], Peter Nilsson[1], Anna Månberg[1*]

1. Department of Protein Science, KTH Royal Institute of Technology, SciLifeLab, Stockholm, Sweden
2. Department of Neurology, University Hospital Ulm (UKU), Ulm, Germany
3. Department of Psychiatry, Technical University of Munich, Munich, Germany
4. kbo-Inn-Salzach-Klinikum gemeinnützige GmbH, Wasserburg am Inn, Germany
5. Department of Neurology, Saarland University, Homburg, Germany
6. Department of Neurodegenerative Diseases and Geriatric Psychiatry, University of Bonn and DZNE Bonn, Bonn, Germany
7. Department of Psychiatry, University Hospital Hamburg, Germany
8. Department of Psychiatry, Friedrich-Alexander University Erlangen-Nuremberg, Erlangen, Germany
9. Center for Mental Health, Department of Psychiatry, Psychosomatics and Psychotherapy, University Hospital Würzburg, Würzburg, Germany
10. Department of Neurology, LMU University Hospital, LMU Munich, Munich, Germany
11. German Center for Neurodegenerative Diseases, site Munich, Munich, Germany
12. Munich Cluster for Systems Neurology (SyNergy), Munich, Germany
13. German Center for Neurodegenerative Diseases (DZNE e.V.), Ulm, Germany
14. Rostock University Medical Center and German Center for Neurodegenerative Diseases (DZNE), Rostock, Germany
15. Clinic for Cognitive Neurology, University Clinic Leipzig, and Max Planck Institute for Human Cognitive and Brain Sciences, Leipzig, Germany
16. Department of Psychiatry and Psychotherapy, University Medical Center Goettingen, and DZNE, Goettingen, Germany
17. Neurosciences and Signaling Group, Institute of Biomedicine (iBiMED), Department of Medical Sciences, University of Aveiro, Aveiro, Portugal
18. Department of Neurology, Martin-Luther-University Halle-Wittenberg, Halle (Saale), Germany

Corresponding author: Anna Månberg; anna.manberg@scilifelab.se

Supplementary figures and tables

Supplementary figures are listed first, followed by supplementary tables.

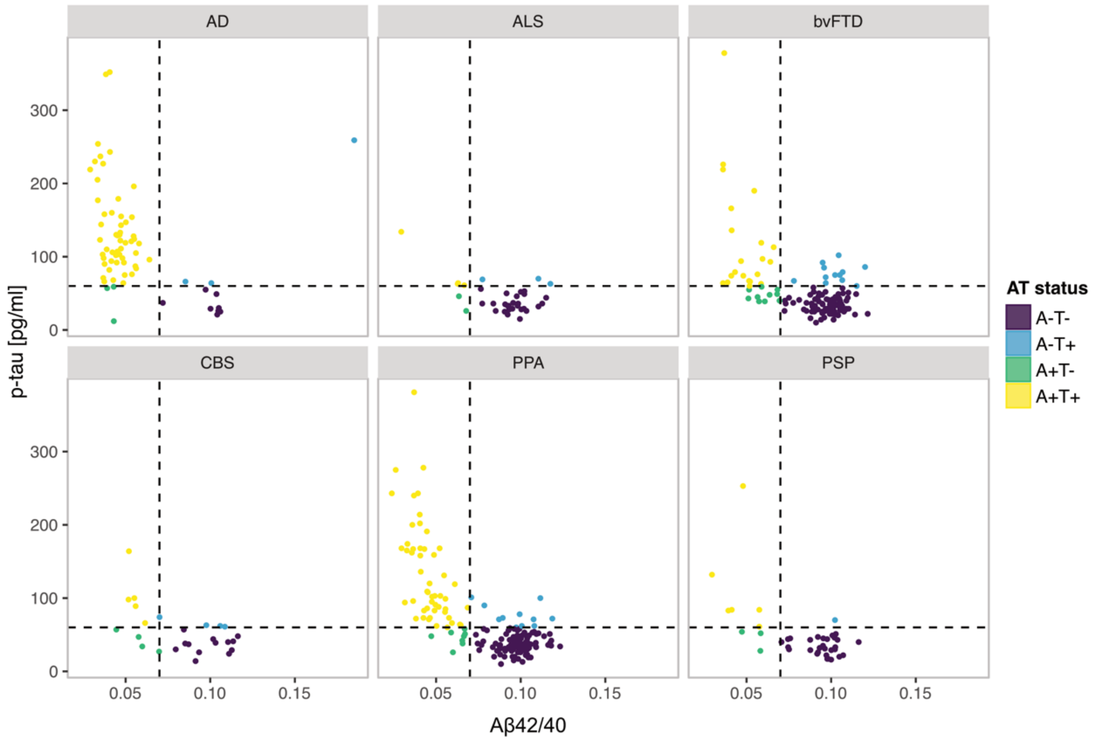

Supplementary figure 1: CSF Aβ42/40 and p-tau levels per diagnosis. The dashed lines label the classification cut-offs: Aβ42/40 = 0.07; p-tau = 60 pg/ml.

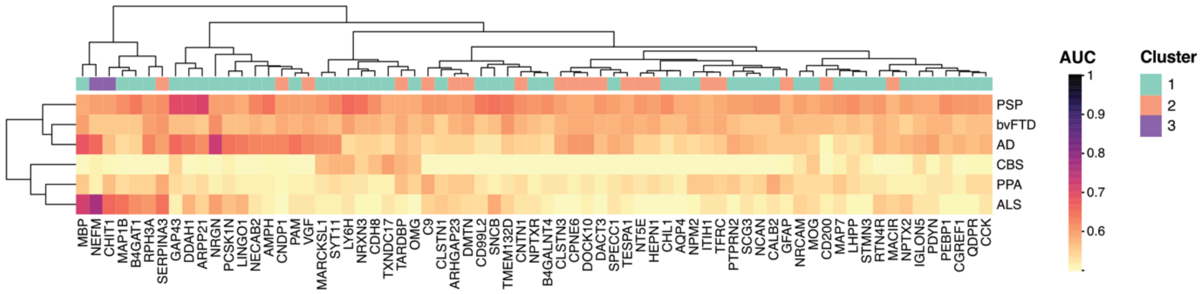

Supplementary figure 2: Disease specificity of CSF proteins. A heatmap showing predictive accuracy (evaluated with AUC) of individual proteins for each disease vs. all diseases, with adjustment for median CSF protein levels. The predicted disease is indicated on the right. The heatmap is annotated on top with the clustering results based on protein correlation (Fig. 1A).

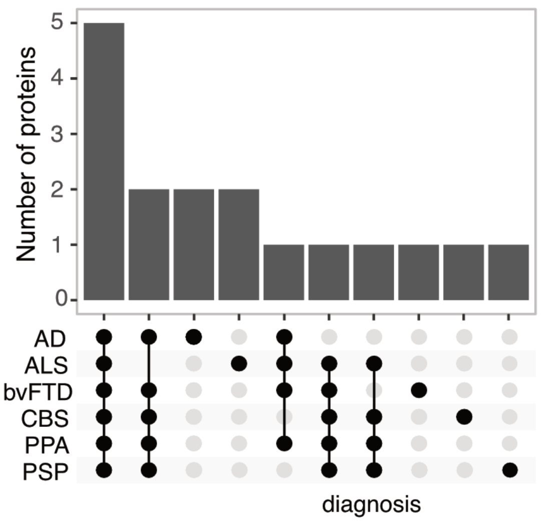

Supplementary figure 3: Top 10 proteins with best performance in classifying disease vs. healthy controls – overlap between diseases

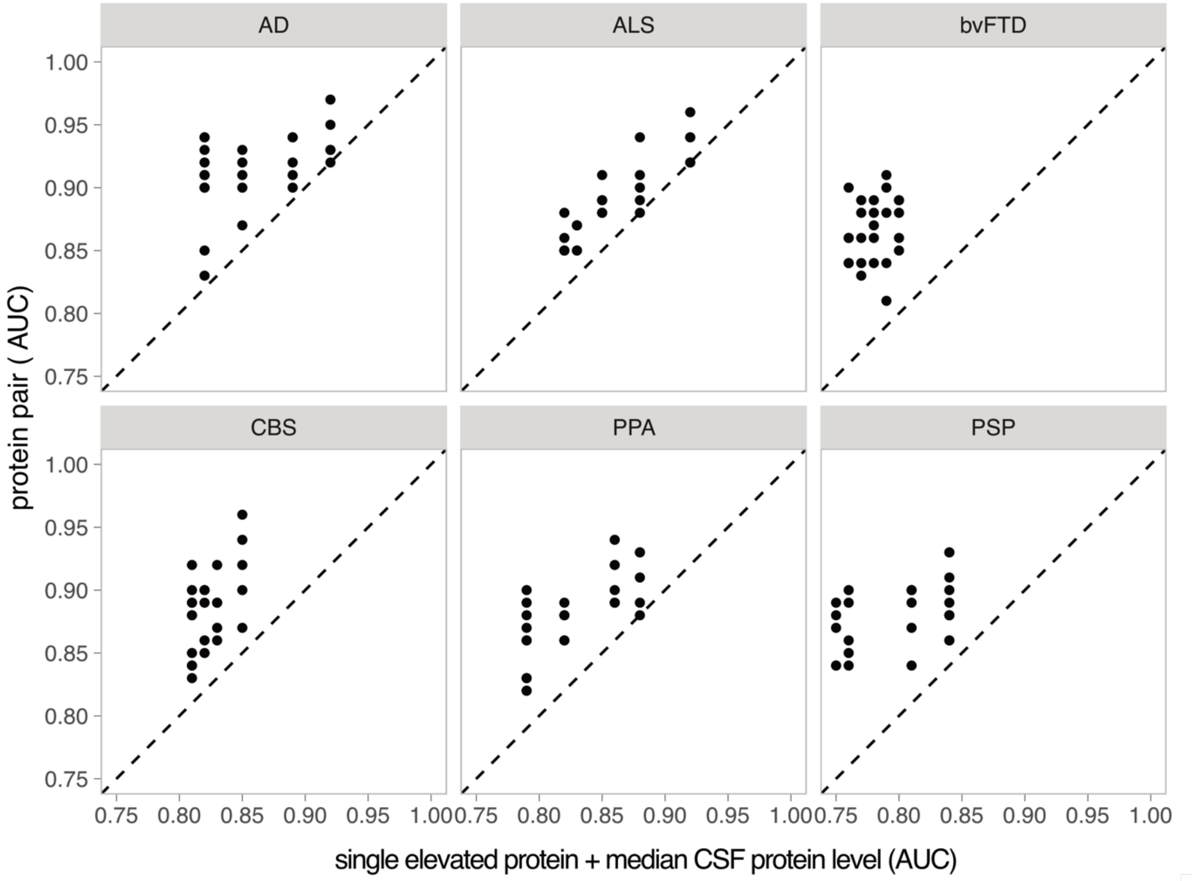

Supplementary figure 4: AUC obtained from logistic regression models for disease versus healthy controls classification. The x-axis represents the AUC values obtained from models with single proteins *elevated* in disease and CSF median protein levels as predictors. On the y-axis, AUC values are depicted for models with protein pairs, where one protein is elevated and one is decreased in disease, as predictors.

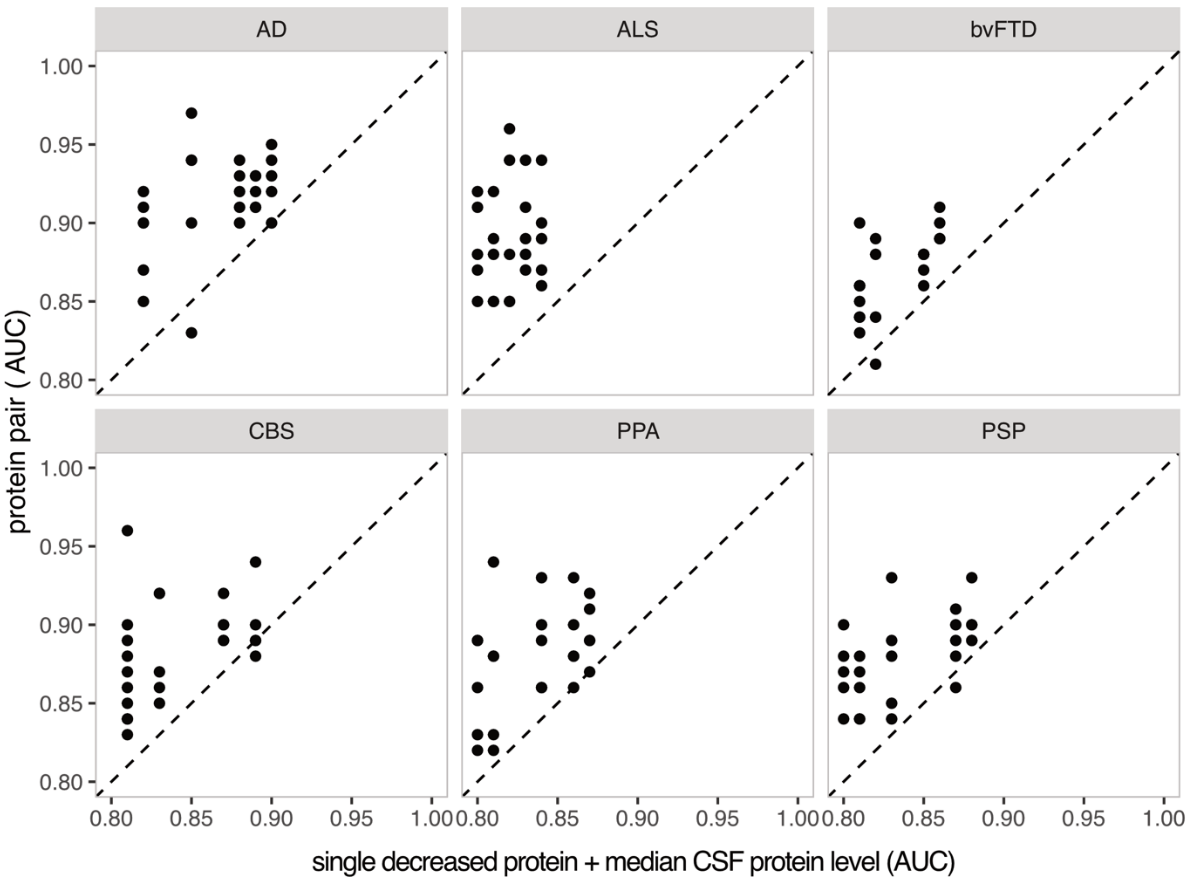

Supplementary figure 5: AUC obtained from logistic regression models for disease versus healthy controls classification. The x-axis represents the AUC values obtained from models with single proteins *decreased* in disease and CSF median protein levels as predictors. On the y-axis, AUC values are depicted for models with protein pairs, where one protein is elevated and one is decreased in disease, as predictors.

Supplementary table 1: List of analysed proteins and used antibodies.

HGNC ID	Protein name	Uniprot ID	Antibody	Sample dilution
**AMPH**	Amphiphysin	P49418	HPA019829	1:25
**AQP4**	Aquaporin 4	P55087	HPA014784	1:200
**ARHGAP23**	Rho GTPase activating protein 23	Q9P227	HPA019818	1:25
**ARPP21**	CAMP regulated phosphoprotein 21	Q9UBL0	HPA017303	1:25
**B4GALNT4**	Beta-1,4-N-acetyl-galactosaminyltransferase 4	Q76KP1	HPA053126	1:25
**B4GAT1**	Beta-1,4-glucuronyltransferase 1	O43505	HPA015484	1:200
**C9**	Complement C9	P02748	HPA070709	1:25
**CALB2**	Calbindin 2	P22676	HPA007305	1:25
**CCK**	Cholecystokinin	P06307	HPA069515	1:200
**CD200**	CD200 molecule	P41217	HPA031149	1:25
**CD99L2**	CD99 molecule like 2	Q8TCZ2	HPA061400	1:200
**CDH8**	Cadherin 8	P55286	HPA014908	1:200
**CGREF1**	Cell growth regulator with EF-hand domain 1	Q99674	HPA008241	1:200
**CHIT1**	Chitinase 1	Q13231	HPA010575	1:25
**CHL1**	Cell adhesion molecule L1 like	O00533	HPA003345	1:200
**CLSTN1**	Calsyntenin 1	O94985	HPA012412	1:200
**CLSTN3**	Calsyntenin 3	Q9BQT9	HPA070830	1:200
**CNDP1**	Carnosine dipeptidase 1	Q96KN2	HPA016933	1:200
**CNTN1**	Contactin 1	Q12860	HPA070467	1:25
**CPNE6**	Copine 6	O95741	HPA031636	1:200
**DACT3**	Dishevelled binding antagonist of beta catenin 3	Q96B18	HPA043053	1:200
**DDAH1**	Dimethylarginine dimethylaminohydrolase 1	O94760	HPA006308	1:25
**DMTN**	Dematin actin binding protein	Q08495	HPA024290	1:200
**DOCK10**	Dedicator of cytokinesis 10	Q96BY6	HPA058106	1:200
**GAP43**	Growth associated protein 43	P17677	HPA013603	1:200
**GFAP**	Glial fibrillary acidic protein	P14136	16825-1-AP	1:25
**HEPN1**	Hepatocellular carcinoma, down-regulated 1	Q6WQI6	HPA063054	1:25
**IGLON5**	IgLON family member 5	A6NGN9	HPA041994	1:25
**ITIH1**	Inter-alpha-trypsin inhibitor heavy chain 1	P19827	HPA042049	1:200
**LHPP**	Phospholysine phosphohistidine inorganic pyrophosphate phosphatase	Q9H008	HPA009269	1:25
**LINGO1**	Leucine rich repeat and Ig domain containing 1	Q96FE5	HPA074653	1:200
**LY6H**	Lymphocyte antigen 6 family member H	O94772	HPA077218	1:25
**MACIR**	Macrophage immunometabolism regulator	Q96GV9	HPA043434	1:25
**MAP1B**	Microtubule associated protein 1B	P46821	HPA022275	1:25
**MAP7**	Microtubule associated protein 7	Q14244	HPA029712	1:25
**MARCKSL1**	MARCKS like 1	P49006	HPA030528	1:25
**MBP**	Myelin basic protein	P02686	HPA049222	1:25
**MOG**	Myelin oligodendrocyte glycoprotein	Q16653	HPA021873	1:200
**NCAN**	Neurocan	O14594	HPA058000	1:200
**NECAB2**	N-terminal EF-hand calcium binding protein 2	Q7Z6G3	HPA013998	1:25
**NEFM**	Neurofilament medium	P07197	HPA022845	1:25
**NPM2**	Nucleophosmin/nucleoplasmin 2	Q86SE8	HPA041070	1:25
**NPTX2**	Neuronal pentraxin 2	P47972	HPA058320	1:200
**NPTXR**	Neuronal pentraxin receptor	O95502	HPA001079	1:25
**NRCAM**	Neuronal cell adhesion molecule	Q92823	HPA061433	1:25

HGNC ID	Protein name	Uniprot ID	Antibody	Sample dilution
**NRGN**	Neurogranin	Q92686	HPA038171	1:25
**NRXN3**	Neurexin 3	Q9HDB5, Q9Y4C0	HPA002727	1:200
**NT5E**	5'-nucleotidase ecto	P21589	HPA048043	1:25
**OMG**	Oligodendrocyte myelin glycoprotein	P23515	HPA008206	1:200
**PAM**	Peptidylglycine alpha-amidating monooxygenase	P19021	HPA042260	1:200
**PCSK1N**	Proprotein convertase subtilisin/kexin type 1 inhibitor	Q9UHG2	HPA064734	1:200
**PDYN**	Prodynorphin	P01213	HPA053342	1:200
**PEBP1**	Phosphatidylethanolamine binding protein 1	P30086	HPA063904	1:200
**PTPRN2**	Protein tyrosine phosphatase receptor type N2	Q92932	HPA007255	1:200
**QDPR**	Quinoid dihydropteridine reductase	P09417	HPA058951	1:25
**RPH3A**	Rabphilin 3A	Q9Y2J0	HPA002475	1:25
**RTN4R**	Reticulon 4 receptor	Q9BZR6	HPA063584	1:200
**SCG3**	Secretogranin III	Q8WXD2	HPA053715	1:200
**SERPINA3**	Serpin family A member 3	P01011	HPA000893	1:200
**SNCB**	Synuclein beta	Q16143	HPA035876	1:200
**SPECC1**	Sperm antigen with calponin homology and coiled-coil domains 1	Q5M775	HPA021421	1:25
**STMN3**	Stathmin 3	Q9NZ72	HPA012947	1:25
**SYT11**	Synaptotagmin 11	Q9BT88	HPA064091	1:25
**TARDBP**	TAR DNA binding protein	Q13148	10782-2-AP	1:25
**TESPA1**	Thymocyte expressed, positive selection associated 1	A2RU30	HPA058823	1:200
**TFRC**	Transferrin receptor	P02786	HPA028598	1:200
**TMEM132D**	Transmembrane protein 132D	Q14C87	HPA010739	1:25
**TXNDC17**	Thioredoxin domain containing 17	Q9BRA2	HPA022931	1:25
**VGF**	VGF nerve growth factor inducible	O15240	HPA058371	1:200

Supplementary table 2: *p*-values from Dunn's test comparing median CSF levels across diagnostic groups

comparison	p-value	adjusted p-value	significant
**AD - ALS**	0.734	0.856	no
**AD - CBS**	0.051	0.133	no
**AD - Healthy control**	0.046	0.160	no
**AD - PPA**	0.286	0.462	no
**AD - PSP**	0.003	0.059	no
**AD - bvFTD**	0.003	0.030	yes
**ALS - CBS**	0.143	0.334	no
**ALS - Healthy control**	0.150	0.316	no
**ALS - PPA**	0.658	0.813	no
**ALS - PSP**	0.023	0.098	no
**ALS - bvFTD**	0.049	0.148	no
**CBS - Healthy control**	0.892	0.937	no
**CBS - PPA**	0.159	0.304	no
**CBS - PSP**	0.557	0.731	no
**Healthy control - PPA**	0.160	0.279	no
**Healthy control - PSP**	0.429	0.601	no
**PPA - PSP**	0.012	0.086	no
**bvFTD - CBS**	0.983	0.983	no
**bvFTD - Healthy control**	0.872	0.964	no
**bvFTD - PPA**	0.013	0.067	no
**bvFTD - PSP**	0.401	0.602	no

Supplementary table 3 and supplementary table 4:

Supplementary table 3: Top five elevated and decreased proteins per disease with the best performance in separating the affected individuals from healthy controls when adjusted for median CSF protein levels.

	**AD**		**ALS**		**CBS**		**PPA**		**PSP**		**bvFTD**	
	**protein**	**AUC**	**protein**	**AUC**	**protein**	**AUC**	**protein**	**AUC**	**protein**	**AUC**	**protein**	**AUC**
**elevated in disease**	GAP43	0.92	NEFM	0.92	NEFM	0.85	NEFM	0.88	MARCKSL1	0.84	GAP43	0.8
	AMPH	0.89	MAP1B	0.88	GAP43	0.83	GAP43	0.86	NEFM	0.84	MAP1B	0.79
	ARPP21	0.85	SNCB	0.85	MARCKSL1	0.82	AMPH	0.82	CALB2	0.81	AMPH	0.78
	MAP1B	0.82	AMPH	0.83	LHPP	0.81	MAP1B	0.79	GAP43	0.76	MARCKSL1	0.77
	NRGN	0.82	MARCKSL1	0.82	AMPH	0.81	MARCKSL1	0.79	AMPH	0.75	QDPR	0.76
**decreased in disease**	PCSK1N	0.9	LINGO1	0.84	LINGO1	0.89	PCSK1N	0.87	LINGO1	0.88	LINGO1	0.86
	LINGO1	0.89	PCSK1N	0.83	PCSK1N	0.87	LINGO1	0.86	PCSK1N	0.87	PCSK1N	0.85
	PDYN	0.88	PDYN	0.82	PTPRN2	0.83	PDYN	0.84	PDYN	0.83	PTPRN2	0.82
	PTPRN2	0.85	NPTX2	0.81	PDYN	0.81	PTPRN2	0.81	CCK	0.81	PDYN	0.81
	CCK	0.82	PTPRN2	0.8	CCK	0.81	CCK	0.8	PTPRN2	0.8	CCK	0.81

Supplementary table 4: Number of individuals per AT group and diagnosis.

	**AT status**			
**Diagnosis**	A+T+	A+T-	A-T+	A-T-
AD	55	3	3	8
ALS	4	2	3	26
CBS	5	4	4	13
PPA	51	8	11	96
PSP	6	3	1	29
bvFTD	21	10	12	86
**total**	142	30	34	258
